# Impact of Birth Control Measures on the Quality of Life Among Married Women

**DOI:** 10.7759/cureus.81595

**Published:** 2025-04-02

**Authors:** Neha Singla, Jasdeep K Jyoti, Nidhi Sagar

**Affiliations:** 1 College of Nursing, All India Institute of Medical Sciences, Raebareli, Raebareli, IND; 2 College of Nursing, Dayanand Medical College and Hospital, Ludhiana, IND

**Keywords:** birth control measures, methods of contraception, oral contraceptives, quality of life, reproductive age group, women, women’s health

## Abstract

Background: Birth control measures are crucial for women's reproductive health and overall well-being. Effective contraception reduces the risk of unintended pregnancies, potentially improving quality of life. However, in India, utilization of birth control remains suboptimal due to various factors, including concerns about side effects and limited access to information. This study investigates the association between birth control measures and quality of life among married women in Punjab, India.

Objectives: The primary aim of the study was to assess the birth control measures and quality of life among married women. Furthermore, to find out the association of birth control measures with selected socio-demographic variables.

Methods: This cross-sectional study enrolled 100 married women of reproductive age (18-45 years) attending antenatal and pediatric outpatient departments at a tertiary care hospital in Punjab. Data collection included socio-demographic characteristics, a clinical profile with birth control measures usage, and a structured five-point Likert scale to assess the quality of life of married women. The collected data was analyzed using IBM SPSS Statistics for Windows, Version 23 (Released 2015; IBM Corp., Armonk, New York, United States), including both descriptive and inferential statistics.

Results: The study of 100 married women revealed a sample predominantly Sikh (59%), with 41% aged 34-40 years and 40% possessing secondary education. Most participants were homemakers (64%), while their spouses were largely employed (92%). A significant majority were mothers (90%), with two children being the most common family size (51.1%). Condoms were the most frequently used contraceptive method (67%), followed by tubectomy (13%), Copper T (Cu T) (12%), and oral contraceptive pills (OCPs) (8%). Overall, 66% reported excellent quality of life. Significant associations were found between contraceptive type and overall (p = 0.03), physical (p < 0.001), social (p < 0.001), and emotional (p < 0.001) well-being, with OCP users reporting higher scores. Furthermore, contraceptive choice was significantly associated with women's education, religion, socio-economic status, number of children, duration of contraceptive use, source of information, and gynecologist consultation (all p < 0.05). However, no significant association was observed between contraceptive method and sexual quality of life (p = 0.92) or satisfaction with the current method (p = 0.44).

Conclusion: This study demonstrates a complex interplay between socio-demographic factors, contraceptive choices, and quality of life among married women in Punjab. The findings highlight the importance of accessible and tailored family planning counseling and education to empower women in making informed decisions about contraception and improve their overall well-being. Further research is needed to explore the specific reasons behind contraceptive choices and their long-term impact on quality of life.

## Introduction

Birth control measures are an important part of the lives of many women, with needs varying according to age [1]. These measures can be defined as various methods, temporary or permanent, used for the control of unwanted pregnancies and thus reducing their potential complications, which significantly affect women’s general health and quality of life.

Numerous methods of contraception are available, which can be categorized as temporary and permanent methods [2]. Globally, contraceptive use varies significantly. In India, specifically, 56.3% of women utilize some form of birth control, with oral combined pills and sterilization being among the most prevalent methods [3]. In Punjab, the prevalence of contraceptive methods was found to be 60.3% in 2015 [4]; however, the use of birth spacing methods is limited, and son preference continues to have a powerful influence on women’s contraceptive use [5], especially on their use of terminal methods. Women’s quality of life is considered an important health marker, as it is directly linked to population control, better health, education, and economic opportunities that lead to lower fertility rates. When women have access to healthcare, family planning, and education, they are more likely to delay childbirth and have fewer children. Improved gender equality and economic empowerment also contribute to informed reproductive choices. This, in turn, supports sustainable population growth and overall societal well-being. Thus, improving the quality of life and controlling population growth are among the principal objectives of any society.

Patient-centered care has become an explicit goal of our healthcare system, so quality of life has become an important health indicator. However, birth control measures have short- and long-term side effects that may occasionally or adversely affect women’s quality of life [6]. Birth control measures play a crucial role in reproductive health and family planning. Contraceptive use helps prevent unintended pregnancies, reducing health risks associated with closely spaced or high-risk pregnancies. For instance, hormonal contraceptives regulate menstrual cycles and lower the risk of ovarian and endometrial cancers. Long-acting reversible contraceptives (LARCs) provide effective, low-maintenance options for women seeking long-term pregnancy prevention. Rayamajhi et al. (2014) [7] reported that women using contraception methods have a significantly higher quality of life compared to those who do not use any contraception.

There is a notable lack of comprehensive studies focusing on how various birth control measures influence women’s quality of life. This gap is particularly significant in the Indian context, where socio-cultural factors may affect both the use and impact of these methods. This study aimed to assess the quality of life of married women utilizing birth control measures and their association with selected socio-demographic measures.

## Materials and methods

Study design and setting

This study adopted a cross-sectional descriptive design that was conducted within the Antenatal and Pediatric Outpatient Departments (OPDs) of Dayanand Medical College and Hospital, Ludhiana, India, from March 2018 to December 2018.

Participant selection and sampling

The study focused on women aged 18 to 45, a demographic segment crucial to reproductive health discussions. The selection criteria targeted women who had consistently used birth control measures for at least six months prior to the study, excluding those with chronic diseases that could confound the assessment of quality of life. Purposive sampling, a non-probability technique, was employed to select 100 participants. While this method allows for targeted selection based on specific criteria, it's essential to acknowledge its potential limitations in terms of generalizability. Exclusion criteria, such as unwillingness to participate and diagnosed psychiatric diseases, further refined the study's focus.

Ethical consideration

Ethical approval was obtained from the Institutional Ethics Committee (IEC), Dayanand Medical College and Hospital (DMCH/R&D/2018/703), ensuring that the research adhered to established ethical principles. Participants were provided with detailed information about the purpose of the study before obtaining their written informed consent. Participation in the study was entirely voluntary, and participants were also assured of the confidentiality and anonymity of the obtained information.

Sample size

The sample size was calculated using the formula: \[ n = \frac{Z^2 P (1 - P)}{d^2} \]

With a 95% confidence interval (Z = 1.96), an anticipated proportion (P) of 50% of married women using birth control, and a desired precision (d) of 10%, the calculated sample size was 96.04. Rounding up to the nearest whole number resulted in 97. However, to ensure adequate power and account for potential attrition, we fixed the sample size at 100.

Data collection and tool

The data collection was done from April 16 to July 20, 2018. The participants were provided with an information sheet describing the aim of the study. After obtaining consent, the tool was given in the form of handouts to the women attending the OPD. It took almost 20-25 minutes for them to complete the tool.

The tool comprises the following three key components:

Socio-Demographic Profile

This section gathered essential background information, including nine variables such as age, education (both women and spouse), occupation (both women and spouse), religion, habitat, type of family, and socio-economic status.

Clinical Profile With Birth Control Measures Usage

This component delved into the specifics of contraceptive use, encompassing 10 variables, including if they had children, number of children, type of previous delivery, current contraceptive method, duration of using the current method, any other contraceptive method used, if they were satisfied with the current method, duration of using the previous method, reason for discontinuing it, and source of informing regarding usage of birth control measures.

Structured Likert Scale

Participants' quality of life was assessed using a structured five-point Likert scale, designed to evaluate four distinct domains: physical, emotional, social, and sexual. Each domain was explored through five items, yielding a total of 20 questions. Responses ranged from 0 (not at all) to 4 (very much), with intermediate options of 1 (a little bit), 2 (sometimes), and 3 (often). The scale incorporated both positively (9 items) and negatively worded items (11 items). Based on the total score, quality of life was categorized into four levels: excellent (61-80), good (41-60), average (21-40), and poor (0-20).

The content validity of the tool was done by seven experts from the fields of medical-surgical nursing, obstetrics and gynecological nursing, and psychiatric nursing, while Cronbach's alpha (r = 0.93) demonstrated high internal consistency, indicating the tool's reliability. To ensure cultural and linguistic appropriateness, the tool was translated into Hindi and Punjabi, and each version was validated by subject language experts.

Statistical analysis

The collected data was analyzed using IBM SPSS Statistics for Windows, Version 23 (Released 2015; IBM Corp., Armonk, New York, United States), a widely recognized statistical software package. A master data sheet was created to organize and manage the data. Descriptive statistics, including frequency and percentage distributions, were used to summarize the demographic and clinical characteristics of the participants. The chi-square test, a non-parametric statistical technique, was employed to examine associations between categorical variables, with a significance threshold of 0.05.

## Results

Sociodemographic characteristics

The study's socio-demographic outcomes revealed that 41% of women were in the age group of 34-40 years, with the mean age 34 ± 6.1, and 40% of married women and their spouses had secondary or senior secondary education. More than two-thirds (64%) of the women were non-working, while the spouses of most women (92%) were employed. More than half (59%) of the married women were Sikh, residing in rural habitats (58%). The largest proportion of women (48%) belonged to the lower middle class (Table 1).

**Table 1:** Demographic details of the participants

Socio-demographic variables	Frequency (n %) (n = 100)
Age (in years) (mean age 34 ± 6.1)	
19-26	9
27-33	36
34-40	41
>40	14
Educational status (women)	
Illiterate	12
Elementary	22
Secondary and senior secondary	40
Graduation and above	26
Educational status (her spouse)	
Illiterate	2
Elementary	28
Secondary and senior secondary	40
Graduation and above	30
Working status (women)	
Working	36
Non-working	64
Working status (her spouse)	
Working	92
Non-working	8
Religion	
Hindu	37
Sikh	59
Muslim	4
Habitat	
Rural	58
Urban	42
Type of family	
Nuclear	51
Joint	49
Socio-economic status (Kuppuswamy scale 2017)	
Upper class	5
Upper middle class	37
Lower middle class	48
Upper lower class	8
Lower class	2

Birth control measures usage

Table 2 presents the clinical profile of the study participants. The vast majority of women (90%) had children. Among those with children (n=90), the most common number of children was two (51.1%), followed by one child (30%). Most deliveries were normal vaginal deliveries (70%), with a smaller proportion being emergency cesarean sections (lower segment cesarean section (LSCS)) (22.2%) and even smaller proportions of normal vaginal deliveries with aids (2.2%) and elective LSCS (4.4%).

**Table 2:** Clinical profile with birth control measures usage details of participants ^* ^other reasons for discontinuing the previous method (allergy, irritation during coitus, displacement of Cu T) ASHA: Accredited Social Health Activist; LSCS: lower segment cesarean section; Cu T: copper T; OCPs: oral contraceptive pills

Clinical profile	Frequency (n %) (n = 100)
Do you have children (n = 100)	
Yes	90
No	10
Number of children (n = 90)	
1	27 (30)
2	46 (51.1)
3	15 (16.7)
4	2 (2.2)
Type of previous delivery (n = 90)	
Normal vaginal delivery	63 (70)
Normal vaginal delivery with aids	2 (2.2)
Emergency LSCS	20 (22.2)
Elective LSCS	4 (4.4)
Current contraception method	
Condoms	67
Cu T	12
OCPs	8
Tubectomy	13
Duration of using the current method	
<1 year	23
1-2 years	7
2-3 years	17
>4 years	53
Any other method used previously	
Yes	40
No	60
Other methods used (n = 40)	
Condoms	17 (42.5)
Copper T	14 (35)
OCPs	9 (22.5)
Are you satisfied with your current method	
Yes	95
No	5
Duration of using the previous method (n = 40)	
<1 year	16 (40)
1-2 years	15 (37.5)
2-3 years	7 (17.5)
>4 years	2 (5)
Reason for discontinuing the previous method (n = 40)	
Irregular bleeding	6 (15)
Body pain	1 (2.5)
Mood swings	5 (12.5)
^ *^Other	28 (70)
Source of information regarding the use of birth control measures	
Media	47
Partner	9
Friends	3
^#^Others (ASHA workers, doctors, Dai, staff nurses)	41

Condoms represent the dominant contraceptive method, employed by 67% of users. Long-term, permanent methods like tubectomy account for 13%, while reversible methods such as copper intrauterine devices (IUDs) (Copper T (Cu T)) and oral contraceptive pills (OCPs) are utilized by 12% and 8% of individuals, respectively. Notably, over half (53%) of those using contraception have maintained their current method for more than four years.

Forty percent of the women reported using other contraceptive methods previously. Among these women (n = 40), the most common previous methods were condoms (42.5%), Cu T (35%), and OCPs (22.5%). The duration of use of these previous methods varied, with the largest proportion (40%) using them for less than one year. The most common reasons for discontinuing the previous method were "other," like allergy, irritation during coitus, and displacement of Cu T (70%), followed by irregular bleeding (15%) and mood swings (12.5%).

The primary sources of information regarding birth control methods were media (47%) and other (ASHA workers, doctors, Dai, and staff nurses) sources (41%), with smaller contributions from partners (9%) and friends (3%). Ninety-five percent of the women expressed satisfaction with their current contraceptive method, while only 5% were dissatisfied.

The majority of women (66%) reported excellent quality of life scores (61-80), with a mean score of 66.50 ± 2.49. A smaller proportion (31%) reported good quality of life (41-60), with a mean score of 55.56 ± 4.17. Only 3% of the women reported average quality of life (21-40), with a mean score of 39.25 ± 2.06. No women reported poor quality of life (0-20) (Table 3).

**Table 3:** Quality of life of married women using birth control measures Maximum score = 80; minimum score = 0

Quality of life	Score	Frequency n (%)	Mean ± SD	Mean %
Excellent	61-80	66	66.50 ± 2.49	83.12
Good	41-60	31	55.56 ± 4.17	69.45
Average	21-40	3	39.25 ± 2.06	49.06

Table 4 examines the association between different birth control measures and quality of life. Among women using condoms, 48 (71.64%) reported excellent quality of life, compared to six (46.15%) of those who had undergone tubectomy, five (41.66%) using Cu T, and seven (87.50%) using OCPs. A chi-square test revealed a statistically significant association between the type of birth control measure used and quality of life (χ² = 8.490, df = 3, p = 0.03).

**Table 4:** Association between birth control measures and quality of life * significant (p < 0.05) Maximum quality of life score is 80; minimum quality of life score is 0 Cu T: copper T; OCPs: oral contraceptive pills

Birth control measures	n	Quality of life n (%)		X^2^	df	p-value
		Excellent	Good			
Condoms	67	48 (71.64)	19 (28.35)	8.490	3	0.03^*^
Tubectomy	13	6 (46.15)	7 (53.84)			
Cu T	12	5 (41.66)	7 (58.33)			
OCPs	8	7 (87.50)	1 (12.5)			

Table 5 presents the association between birth control measures and different domains of quality of life. Statistically significant associations were found between the type of birth control measure used and the physical (χ² = 24.93, df = 9, p < 0.001), social (χ² = 21.65, df = 9, p < 0.001), and emotional (χ² = 16.11, df = 6, p < 0.001) domains of quality of life. No statistically significant association was observed for the sexual domain (χ² = 6.01, df = 6, p = 0.92).

**Table 5:** Association of birth control measures with different domains of quality of life ^* ^significant (p < 0.05); ^NS ^non-significant (p > 0.05)

Domains	Condom (n = 67)	Cu T (n = 12)	OC pills (n = 8)	Tubectomy (n = 13)	Chi-square (χ2)	df	p-value
								Physical domain					24.93	9	0.00*
Excellent	5 (7.47%)	0	3 (37.5%)	2 (15.38%)											
Good	56 (83.58%)	7 (58.33%)	5 (62.5%)	6 (46.16%)											
Average	5 (7.46%)	4 (33.33%)	0	3 (23.08%)											
Poor	1 (1.49%)	1 (8.34%)	0	2 (15.38%)											
Social domain					21.65	9	0.00*
Excellent	55 (82.09%)	6 (50%)	8 (100)	7 (53.85%)			
Good	10 (14.93%)	5 (41.67%)	0	4 (30.77%)			
Average	2 (2.98%)	0	0	2 (15.38%)			
Poor	0	1 (8.33%)	0	0			
Emotional domain					16.11	6	0.00*
Excellent	61 (91.05%)	10 (83.34%)	6 (75%)	9 (69.24%)			
Good	6 (8.95%)	2 (16.66%)	2 (25%)	2 (15.38%)			
Average	0	0	0	2 (15.38%)			
Sexual domain					6.01	6	0.92^NS^
Excellent	41 (61.20%)	8 (66.67%)	7 (87.5%)	6 (46.15%)			
Good	24 (35.82%)	3 (25%)	1 (12.5%)	7 (53.85%)			
Average	2 (2.98%)	1 (8.33%)	0	0			

Table 6 presents the association between birth control measures and selected socio-demographic characteristics. Statistically significant associations were found between the type of birth control measure used and women's educational status (χ² = 18.60, df = 9, p = 0.02), religion (χ² = 19.14, df = 9, p = 0.00), socio-economic status (χ² = 19.59, df = 9, p = 0.02), and number of children (χ² = 33.79, df = 12, p < 0.001). No statistically significant associations were observed with age, spouse's education, women's working status, spouse's working status, women's occupation, habitat, family status, child status, or type of previous delivery.

**Table 6:** Association of birth control measures with selected socio-demographic characteristics ^* ^significant (p < 0.05); ^NS^ non-significant (p > 0.05); ^# ^some of the categories are combined to apply chi-square NVD: normal vaginal delivery; LSCS: lower segment cesarean section; Cu T: copper intrauterine device (copper T); OC: oral contraceptive

Variables	Condoms (n = 67)	Cu T (n = 12)	OC pills (n = 8)	Tubectomy (n = 13)	Chi square (χ2)	df	p-value
								Age (in years)					10.22	9	0.33^NS^
19-26	8	1	0	0											
26-33	27	5	3	1											
33-40	24	4	4	9											
>40	8	2	1	3											
Educational status (women)					18.6	9	0.02*
Illiterate	5	0	3	4			
Elementary	16	2	1	3			
Secondary and senior secondary	25	9	2	4			
Graduation	21	1	2	2			
Educational status (spouse)					22.62	9	0.07^NS^
Illiterate	0	0	0	2			
Elementary	18	2	5	3			
Secondary and senior secondary	26	6	1	7			
Graduation	23	4	2	1			
Working status (women)					2.51	3	0.47^NS^
Working	23	3	3	7			
Non-working	44	9	5	6			
Working status (spouse)					3.25	3	0.35^NS^
Working	63	10	8	11			
Non-working	4	2	0	2			
Occupation (women)					26.79	9	0.5^NS^
Labour	8	0	0	4			
Private job	11	1	0	0			
Government job	5	3	3	1			
Agriculture	0	2	0	2			
Religion					19.14	9	0.00*
Hindu	27	2	3	5			
Sikh	40	8	3	8			
Muslims	0	2	2	0			
Habitat					4.2	3	0.24^NS^
Rural	35	8	7	8			
Urban	32	4	1	5			
Family status					5.74	3	0.12^NS^
Joint	34	6	6	3			
Nuclear	33	6	2	10			
^#^Socio-economic status					19.59	9	0.02*
Upper class	3	1	1	0			
Upper middle class	26	6	2	3			
Lower middle class	35	5	2	6			
Upper lower class	3	0	3	4			
Child status					5.05	3	0.16^NS^
Yes	59	12	6	13			
No	8	0	2	0			
Number of children					33.79	12	0.00*
0	8	5	2	0			
1	21	5	1	2			
2	30	6	4	6			
>3	10	1	2	5			
Type of previous delivery					11.71	9	0.23^NS^
Normal vaginal delivery	41	6	5	11			
NVD with aids	3	0	1	0			
Emergency LSCS	14	4	1	1			
Elective LSCS	1	2	0	1			

Table 7 presents the association between birth control measures and clinical profile. Statistically significant associations were found between the type of birth control method used and the duration of using the current method (χ² = 26.20, df = 9, p = 0.02), the source of information about birth control (χ² = 43.11, df = 9, p < 0.001), and whether the woman had consulted a gynecologist (χ² = 48.28, df = 3, p < 0.001). No statistically significant association was found between satisfaction with the current method and the type of birth control used (χ² = 2.67, df = 3, p = 0.44).

**Table 7:** Association between birth control measures and clinical profile * significant (p < 0.05); ^NS^ non-significant (p > 0.05) OC: oral contraceptive

Variables					Chi-square (χ^2^)	df	p-value
	Condom (n = 67)	Copper T (n = 12)	OC pills (n = 8)	Tubectomy (n = 13)			
Duration of using current method					26.2	9	0.02^*^
<1 year	15	3	4	1			
1-2 years	4	1	0	2			
2-3 years	8	6	3	0			
≥4 years	40	2	1	10			
Are you satisfied with the current method?					2.67	3	0.44^NS^
Yes	60	12	8	13			
No	5	0	0	0			
Source of information					43.11	9	0.00^*^
Media	42	1	4	0			
Partner	8	0	1	0			
Friends	3	0	0	0			
Others	14	11	3	13			
Have you consulted your gynecologist?					48.28	3	0.00^*^
Yes	8	11	7	9			
No	58	1	1	4			

## Discussion

Quality of life has been defined as an individual's perceptions of their position in life in the context of the culture and value systems in which they live and in relation to their goals, expectations, standards, and concerns (WHO) [8].

The findings of the present study indicate that the majority of married women (67%) were using condoms, 13% had undergone tubectomy, 12% were using Cu T, and 8% were using OCPs. This conclusion is supported by Shah et al. (2018), who found that among 82 women, 70.7% used male condoms, 11.0% used OCPs, 8.5% used IUDs, and 4.9% used implants [9].

In the present study, certain factors significantly influence the choice of birth control methods among married women, like educational status (p = 0.02), religion (p = 0.00), socio-economic status (p = 0.02), and number of children (p = 0.00), and in the clinical profile, duration of using the current method (p = 0.02), source of information (p = 0.00), and consultation with a gynecologist (p = 0.00). These results align with Nair et al. (2016) [10], which found a significant link between education and information sources with contraceptive use.

Age is significantly associated with contraceptive use, with women aged 34-40 being 41% more likely to use modern contraceptives and women aged 27-33 being 36% more likely, compared to those aged 19-26 being 9% only. This pattern is consistent with studies from Ethiopia [11] and Bangladesh [12], which show that older women are more likely to use contraception, as they may have completed or nearly completed their family size [13]. However, a study in Cameroon [14] shows that younger women, particularly those still in school, tend to use contraception more to delay family planning and focus on education.

The present study revealed that health professionals and the media were the primary sources of information regarding contraceptive methods. This finding aligns with the study conducted by Alduraywish et al. [15], highlighting the crucial role of these sources in shaping women's contraceptive choices.

A strong positive association between birth control use and quality of life was observed, with 66% of women reporting excellent well-being. Condom use was particularly associated with positive outcomes, as 71.64% of these women reported an excellent quality of life (p = 0.02), mirroring the results of Shah et al. (2018) [9], who found a similar correlation (p = 0.01). OCPs have also been shown to improve the quality of life in some women, as evidenced by Zhao et al.'s (2009) [16] study of rural Chinese women using oral pills and IUDs.

Several factors may explain these findings. The higher quality of life scores among condom users could be attributed to the method's non-hormonal nature, minimizing potential side effects that can negatively impact well-being. Additionally, the control offered by condoms in preventing sexually transmitted infections (STIs) may contribute to a sense of security and improved psychological health. OCP users also reported high quality of life, potentially due to the method's effectiveness and the control it provides over fertility.

The lower proportion of excellent quality of life among women who had undergone tubectomy or used Cu T could be related to various aspects. Tubectomy, while offering permanent contraception, is a surgical procedure that may be associated with postoperative discomfort or psychological adjustments. Cu T, while effective, can sometimes cause side effects like heavier menstrual bleeding or pelvic pain, potentially affecting quality of life.

While our study, alongside others, points toward a positive association between contraceptive use and quality of life, it's essential to acknowledge the potential for negative impacts and address the complexities surrounding this issue. As Alspaugh et al. [17] highlighted in their review of qualitative studies, concerns regarding cancer risk and fertility are frequently raised. The relationship between contraceptive use and cancer risk is far from straightforward. For instance, while some studies have suggested a potential increase in the risk of certain cancers, such as breast cancer in some hormonal contraceptive users [18], others have demonstrated a decreased risk for other cancers, like endometrial and ovarian cancers [19]. Our study didn't directly examine these concerns, yet their influence on contraceptive decisions and perceived quality of life cannot be understated. Future investigations should prioritize detailed exploration of these anxieties, including measures of perceived risk and associated anxiety.

The strength of the study lies in the adequacy of a statistically appropriate sample size, enhancing the reliability of the findings within this specific demographic. Secondly, it provides representation across various contraceptive methods, allowing for comparative analyses of their effects on quality of life.

Limitations of the study

This study is limited by its small, purposive sample of 100 women from specific clinics, potentially limiting generalizability. Future research employing larger, more representative samples, longitudinal designs, and potentially incorporating objective measures of quality of life would be beneficial.

Recommendation for practice

There exists a need to develop educational materials to inform women about the potential effects of birth control on quality of life and to support informed decision-making. Thorough counseling on potential side effects of different contraceptive methods can be provided to women, which will further encourage shared decision-making between women and their healthcare providers regarding birth control options. Implement targeted interventions for diverse demographics, ensure ongoing provider training, and conduct further research on long-term impacts to optimize women's well-being.

## Conclusions

This study provides valuable insights into the complex relationship between contraceptive choices and quality of life for married women. While our findings suggest a generally positive association, the specific impact of birth control on well-being is nuanced and varies depending on the chosen method. Recognizing the multifaceted nature of quality of life, future research should prioritize investigating the interplay between health status, sociodemographic factors, social circumstances, and contraceptive use. A deeper understanding of these intricate relationships will be essential for developing targeted interventions that empower women to make informed decisions about their reproductive health, ultimately improving their overall quality of life.

## References

[1] (2023). Very well health. Fact sheet: an overview of birth control. https://www.verywellhealth.com/birth-control-4014756.

[2] Konar H (2016). Textbook of Obstetrics. https://hiralalkonar.com/images/Konar%20-%20Obstetrics-blad-9th.pdf.

[3] (2023). National Family Health Survey (NFHS-5). https://mohfw.gov.in/sites/default/files/NFHS-5_Phase-II_0.pdf.

[4] Umran O, Melike D (2016). Effect of the contraceptive methods on female sexual function. Int J Caring Sci.

[5] Malmborg A, Persson E, Brynhildsen J, Hammar M (2015). Hormonal contraception and sexual desire: a questionnaire-based study of young Swedish women. Eur J Contracept Reprod Health Care.

[6] Bahri N, Tohidinik HR, Bilandi RR, Larki M, Hooshangi F, Soltanian M (2016). The relation between contraception methods and quality of life. Epidemiol Biostat Public Health.

[7] Rayamajhi RB, Paudel IS, Bhattarai S, Neupane B, Pokharel PK (2014). Practice of contraception and quality of life among Bhutanese refugee women of eastern Nepal. Glob J Med Res K Interdiscip.

[8] (2019). WHOQOL - brief introduction, administration, scoring and generic version of the assessment. http://www.who.int/mental_health/media/en/76.pdf.

[9] Shah R, Kiriya J, Shibanuma A, Jimba M (2018). Use of modern contraceptive methods and its association with QOL among Nepalese female migrants living in Japan. PLoS One.

[10] Nair RV, Ashok VG, Solanke PV (2016). A study on contraceptive use among married women of reproductive age group in a rural area of Tamilnadu, India. Int J Reprod Contracept Obstet Gynecol.

[11] Gebre MN, Edossa ZK (2020). Modern contraceptive utilization and associated factors among reproductive-age women in Ethiopia: evidence from 2016 Ethiopia demographic and health survey. BMC Womens Health.

[12] Haq I, Sakib S, Talukder A (2017). Sociodemographic factors on contraceptive use among ever-married women of reproductive age: evidence from three demographic and health surveys in Bangladesh. Med Sci (Basel).

[13] Sedgh G, Hussain R (2014). Reasons for contraceptive nonuse among women having unmet need for contraception in developing countries. Stud Fam Plann.

[14] Njotang PN, Yakum MN, Ajong AB (2017). Determinants of modern contraceptive practice in Yaoundé-Cameroon: a community based cross sectional study. BMC Res Notes.

[15] Alduraywish SA, Altamimi LA, Aldhuwayhi RA (2020). Sources of health information and their impacts on medical knowledge perception among the Saudi Arabian population: cross-sectional study. J Med Internet Res.

[16] Zhao J, Li Y, Wu Y (2009). Impact of different contraceptive methods on quality of life in rural women of the Jiangsu province in China. Contraception.

[17] Alspaugh A, Barroso J, Reibel M, Phillips S (2020). Women’s contraceptive perceptions, beliefs, and attitudes: an integrative review of qualitative research. J Midwifery Womens Health.

[18] Mørch LS, Skovlund CW, Hannaford PC, Iversen L, Fielding S, Lidegaard Ø (2017). Contemporary hormonal contraception and the risk of breast cancer. N Engl J Med.

[19] MacKintosh ML, Crosbie EJ (2018). Prevention strategies in endometrial carcinoma. Curr Oncol Rep.

